# Ring fencing of a jount replacement unit may not prevent MRSA in practice

**DOI:** 10.1186/1753-6561-5-S6-P198

**Published:** 2011-06-29

**Authors:** H-MA Schmidt, C Izon, MW Maley

**Affiliations:** 1 Microbiology and Infectious Diseases, Sydney South West Pathology Service, Liverpool Hospital, Sydney, Australia; 2 Infection Control, Fairfield Hospital, Sydney, Australia

## Introduction / objectives

Ring fencing of joint replacement (JR) surgery units is recommended to prevent the high morbidity and mortality associated with methicillin resistant *Staphylococcus aureus* (MRSA) infection in orthopaedic patients. However, little is known about the effectiveness of and compliance with such policies in practice.

## Methods

Over 10 weeks in 2010, 250 prospectively recruited admissions to a busy, ring fenced JR unit underwent admission screening for MRSA and demographic screening using a standardised questionnaire based on the unit’s admission policy which is designed to exclude patients at high risk of MRSA colonisation. Subjects comprised of patients admitted for reasons other than JR surgery, as well as JR patients who stayed in any other hospital ward prior to admission to the unit.

## Results

Despite nearly perfect compliance (248/250; 99.2%) with unit’s admission policy, 2.8% (7/248) of subjects complying with the admission policy were colonised with MRSA at the nares and/or groin. MRSA carriers were disparate in age, gender and inpatient hospital admission in the last 12 months. Non-JR patients and transfers from high dependency units represented a high risk for introducing MRSA into a ring fenced unit.

## Conclusion

Demographic screening undoubtedly excludes a proportion of MRSA carriers; however, it is inadequate to completely prevent the admission of MRSA to a ring fenced unit. Where it is unavoidable that ring fenced units host patients not admitted for JR surgery and not screened for MRSA preadmission, these patients should be cohorted away from JR patients as they represent a higher risk for MRSA despite rigorous demographic screening.

## Disclosure of interest

None declared.

